# Chromatographic Separation: A Versatile Strategy to Prepare Discrete and Well-Defined Polymer Libraries

**DOI:** 10.1021/acs.accounts.4c00059

**Published:** 2024-03-26

**Authors:** Elizabeth A. Murphy, Cheng Zhang, Christopher M. Bates, Craig J. Hawker

**Affiliations:** ^†^Materials Research Laboratory, ^‡^Department of Chemistry & Biochemistry, ^§^Department of Chemical Engineering, and^#^Materials Department, University of California Santa Barbara, Santa Barbara, California 93106, United States; ⊥ Australian Institute for Bioengineering and Nanotechnology and Centre for Advanced Imaging University of Queensland, Brisbane, Queensland 4072, Australia

## Abstract

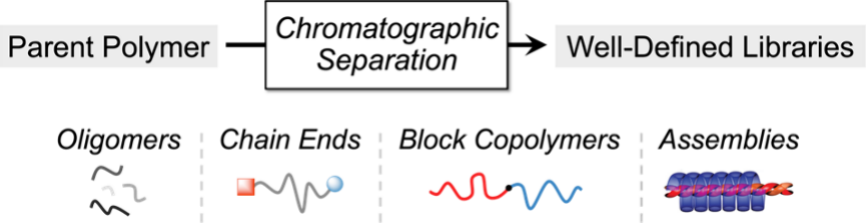

The preparation of discrete and well-defined polymers is an emerging strategy for emulating the remarkable precision achieved by macromolecular synthesis in nature. Although modern controlled polymerization techniques have unlocked access to a cornucopia of materials spanning a broad range of monomers, molecular weights, and architectures, the word “controlled” is not to be confused with “perfect”. Indeed, even the highest-fidelity polymerization techniques—yielding molar mass dispersities in the vicinity of *Đ* = 1.05—unavoidably create a considerable degree of structural and/or compositional dispersity due to the statistical nature of chain growth. Such dispersity impacts many of the properties that researchers seek to control in the design of soft materials.

The development of strategies to minimize or entirely eliminate dispersity and access molecularly precise polymers therefore remains a key contemporary challenge. While significant advances have been made in the realm of iterative synthetic methods that construct oligomers with an exact molecular weight, head-to-tail connectivity, and even stereochemistry via small-molecule organic chemistry, as the word “iterative” suggests, these techniques involve manually propagating monomers one reaction at a time, often with intervening protection and deprotection steps. As a result, these strategies are time-consuming, difficult to scale, and remain limited to lower molecular weights. The focus of this Account is on an alternative strategy that is more accessible to the general scientific community because of its simplicity, versatility, and affordability: chromatography. Researchers unfamiliar with the intricacies of synthesis may recall being exposed to chromatography in an undergraduate chemistry lab. This operationally simple, yet remarkably powerful, technique is most commonly encountered in the purification of small molecules through their selective (differential) adsorption to a column packed with a low-cost stationary phase, usually silica. Because the requisite equipment is readily available and the actual separation takes little time (on the order of 1 h), chromatography is used extensively in small-molecule chemistry throughout industry and academia alike. It is, therefore, perhaps surprising that similar types of chromatography are not more widely leveraged in the field of polymer science as well.

Here, we discuss recent advances in using chromatography to control the structure and properties of polymeric materials. Emphasis is placed on the utility of an adsorption-based mechanism that separates polymers based on polarity and composition at tractable (gram) scales for materials science, in contrast to size exclusion, which is extremely common but typically analyzes very small quantities of a sample (∼1 mg) and is limited to separating by molar mass. Key concepts that are highlighted include (1) the separation of low-molecular-weight homopolymers into discrete oligomers (*Đ* = 1.0) with precise chain lengths and (2) the efficient fractionation of block copolymers into high-quality and widely varied libraries for accelerating materials discovery. In summary, the authors hope to convey the exciting possibilities in polymer science afforded by chromatography as a scalable, versatile, and even automated technique that unlocks new avenues of exploration into well-defined materials for a diverse assortment of researchers with different training and expertise.

## Key References

- LawrenceJ.; LeeS.-H.; AbdillaA.; NothlingM. D.; RenJ. M.; KnightA. S.; FleischmannC.; LiY.; AbramsA. S.; SchmidtB. V. K. J.; HawkerM. C.; ConnalL. A.; McGrathA. J.; ClarkP. G.; GutekunstW. R.; HawkerC. J.A Versatile and Scalable Strategy to Discrete Oligomers. J. Am. Chem. Soc.2016, 138 ( (19), ), 6306–631027152711 10.1021/jacs.6b03127PMC4879877.^1^ This work demonstrates a versatile strategy for the multigram synthesis of a broad range of discrete oligomers using a combination of automated chromatographic separation and controlled polymerization.
- LawrenceJ.; GotoE.; RenJ. M.; McDearmonB.; KimD. S.; OchiaiY.; ClarkP. G.; LaitarD.; HigashiharaT.; HawkerC. J.A Versatile and Efficient Strategy to Discrete Conjugated Oligomers. J. Am. Chem. Soc.2017, 139 ( (39), ), 13735–1373928872865 10.1021/jacs.7b05299.^2^ This work expands the available substrate scope of discrete materials to conjugated oligomers. Using this separation strategy, chain-length physical, optical, and electronic properties were elucidated.
- ZhangC.; BatesM. W.; GengZ.; LeviA. E.; VigilD.; BarbonS. M.; LomanT.; DelaneyK. T.; FredricksonG. H.; BatesC. M.; WhittakerA. K.; HawkerC. J.Rapid Generation of Block Copolymer Libraries Using Automated Chromatographic Separation. J. Am. Chem. Soc.2020, 142 ( (21), ) 9843–984932421319 10.1021/jacs.0c04028.^3^ This works reported the rapid generation of block copolymer libraries spanning a wide range of compositions and morphologies starting from a single parent copolymer.
- MurphyE. A.; ChenY.-Q.; AlbaneseK.; BlankenshipJ. R.; AbdillaA.; BatesM. W.; ZhangC.; BatesC. M.; HawkerC. J.Efficient Creation and Morphological Analysis of ABC Triblock Terpolymer Libraries. Macromolecules2022, 55 ( (19), ), 8875–8882.^4^ This work expands the chromatographic fractionation of block copolymers to complex, high-molecular-weight ABC triblock terpolymers. Fractionation improves the long-range order of nanoscale morphologies compared to as-synthesized materials and accelerates discovery over a multidimensional design space.

## 1 Introduction

Nature has long mastered the synthesis and use of well-defined macromolecules in biology. For example, natural polymers such as DNA, RNA, proteins, and nucleic acids^5^ can be perfectly monodisperse (*Đ* = 1.00) in size, composition, sequence, and chirality,^6^ leading to control over important biological functions spanning molecular recognition,^7^ catalysis,^8^ information storage,^9^ and selective transport.^10^ While this level of structural specificity remains out of reach with synthetic polymers due to the statistical nature of polymerization processes, these principles can drive important directions in polymer synthesis. Inspired by this difference between natural and synthetic systems, a long-standing “grand challenge” is the development of strategies to precisely control the structure of polymers.^11^ This lofty goal is not just motivated by a desire to refine our collective synthetic toolkit; advances would impact the fundamental understanding of many types of dispersity, and nearly all of them are known to impact the properties of polymeric materials, from mechanics^12−15^ to nanostructure^16−19^ and optics.^2^

Significant advances have been made in preparing precise polymers through stepwise strategies such as solid-phase peptide (Merrifield) synthesis^20−23^ and iterative exponential growth.^24−27^ Both require the sequential addition of individual monomers through a sequence of chemical reactions, often with purification between steps. Unlike classical polymerization, these methods are generally time-consuming, limited in scale, required to be reoptimized for different monomers, and restricted to low molecular weights. Consequently, they are especially challenging for nonexperts to employ.^28^

The emergence of controlled polymerization techniques (e.g., atom transfer radical polymerization (ATRP) and reversible addition–fragmentation chain-transfer polymerization (RAFT)) has greatly improved the availability of well-defined polymers on large scales.^29,30^ Conceptually, these approaches provide better control over average chain length, molecular weight dispersity, end-group fidelity, and architecture while retaining the scalability of traditional (uncontrolled) polymerization techniques.^31^ Nevertheless, the term “controlled” should not be misconstrued: the resulting polymers still exhibit substantial structural and compositional dispersity.^32^ As a result, simple methods to prepare discrete synthetic polymers by direct polymerization strategies currently do not exist due to the stochastic nature of initiation, propagation, and termination. The development of versatile, efficient, and scalable strategies to realize discrete and well-defined polymers libraries therefore stands as a key contemporary challenge.

As an alternative to directly synthesizing precise polymers, there is growing interest in post-polymerization purification aimed at achieving novel, narrow-dispersity materials. An attractive option is chromatography, a well-known technique in organic chemistry.^33^ Broadly speaking, the separation mechanism either distinguishes fractions by hydrodynamic volume (“size exclusion”), chemical affinity (adsorption), or some combination thereof. Although extensively used in the purification of small molecules, chromatography is significantly less common as a preparatory separation tool in polymer science. A few pioneering reports have hinted at the potential utility including the isolation of discrete poly(ethylene oxide)^34^ and poly(styrene) oligomers^35^ and the preliminary separation of poly(styrene)-based block polymers.^36−40^ A common thread among these initial examples is the complexity in experimental design, including carefully choosing sample and solvent compatibility, temperature, detector configuration(s), and injection sequence.^41−43^ In part because of such complexity, only specialist groups have used these chromatography experiments generally on analytical scales (∼100 mg or less). Clearly, the promise of coupling scalable controlled polymerization techniques with the precision of chromatographic separation is tempered if these strategies are only available to experts and on small scales.

In this Account, we present a comprehensive overview of our group’s contributions to the development of automated chromatography as an efficient and scalable approach for nonexperts to generate well-defined polymer libraries. Initially, we highlight the crucial role of the commercially available automated instrumentation to afford well-defined materials. We then discuss our efforts to use automated chromatography for preparing discrete oligomers (*Đ* = 1.00) on multigram scales across a broad range of monomer families. We further examine the use of automated chromatography to rapidly build libraries of high-molecular-weight multiblock copolymers from controlled polymerization processes. Finally, we spotlight achievements by other researchers, where this large and growing body of work collectively underscores the accessibility and versatility of automated chromatography coupled with controlled polymerization and its potential to facilitate the discovery of novel, well-defined advanced materials with unique and useful properties.

## 2 Automated Chromatography for Polymeric Systems

**Figure 1:**
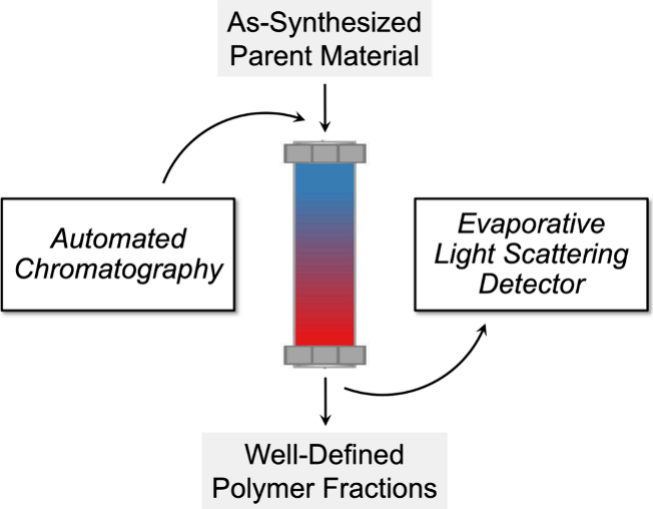
Schematic diagram of automated chromatographic separation, which separates polymers through an adsorption-based mechanism, typically with a solvent gradient determined by routine TLC analysis. Different detector configurations are possible with evaporative light scattering being particularly powerful for universal materials detection.

An ideal method for creating well-defined polymeric libraries would be user-friendly and leverage common laboratory equipment that is simple to use and broadly available. As introduced above, one appealing technique that satisfies these ideals is column chromatography. This simple and inexpensive toolkit enables the precise and reproducible separation of complex polymer mixtures using standardized protocols—crucial for generating reliable data and facilitating detailed comparisons between samples and across different experiments. This type of chromatography separates based on differential chemical affinity (adsorption) to a stationary phase (e.g., silica), in contrast to other mechanisms such as size exclusion used in gel permeation chromatography—a more prevalent method in polymer science. An adsorption-based mechanism proves particularly valuable when separating complex mixtures of disperse polymer chains with varying compositions stemming from the statistical nature of polymerization processes. Unlike size exclusion, this approach facilitates the separation of polymer chains from an as-synthesized parent mixture based on constituent polarity, providing new methods for preparing polymer libraries with enhanced structural precision (Figure 1).

**Figure 2:**
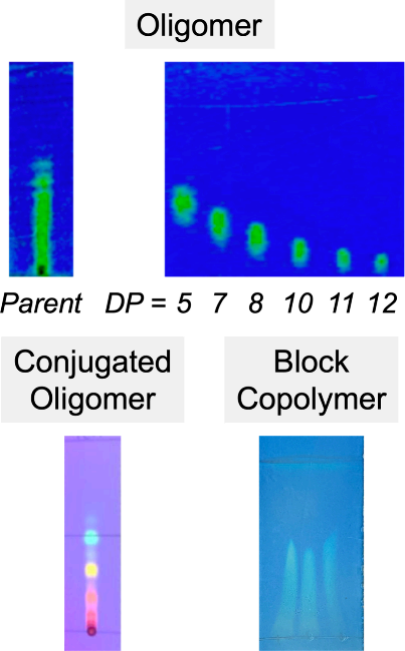
Thin-layer chromatography rapidly yields optimized solvent conditions for automated chromatography with a diverse range of materials. A streaking pattern indicates favorable elution conditions that are conducive to efficient compositional fractionation. Reproduced with permission from refs (1, 2, and 4). Copyright 2017, 2016, and 2022 American Chemical Society.

In practice, commercially available column cartridges are used in a variety of sizes (e.g., 5–350 g) with different average particle diameters (e.g., 20–60 μm), pore dimensions (e.g., 100–300 Å), loading capacities, and surface functionalization (e.g., normal phase, C_18_-functionalized, amine-functionalized). The mass and chemistry of a given sample being separated determine the appropriate choice of column conditions, with insights drawn from analogous small-molecule purification. Similar to chromatography of small molecules, sufficient polarity differences between polymeric constituents are needed for efficient separation. Achieving a diverse library of well-defined polymers with varying compositions necessitates a balance between solvent strength and gradient profile. Preliminary thin-layer chromatography (TLC) experiments prior to larger-scale separation are used to identify optimal solvent conditions (Figure 2). A few key observations are a simple strategy for identifying initial TLC experiments to an optimal solvent gradient—two or more cosolvents varied in their ratio throughout the separation process—for the preparation of high-quality polymeric libraries. First, an ideal weak solvent should solubilize the polymer but not result in appreciable mobility from the baseline of a TLC plate (*R*_f_ value ∼ 0). Second, mixing the weak solvent with a more polar solvent yields an optimized solvent pair ratio when it causes streaking of the polymer across a TLC plate, indicating well-resolved separation between constituents with varying polarity (i.e., chain length and/or composition) from the disperse mixture. This distinct streaking pattern represents a promising elution condition that is conducive to efficient compositional fractionation at larger scales.

After determining an appropriate solvent pair, gradient profiles are optimized to control the speed of polymer elution. The solvent strength should be programmed to gradually increase (e.g., linearly increase) to achieve incremental compositional changes of the polymer, a process that is greatly facilitated by the use of automation. Throughout the separation process, polymer elution can be carefully monitored by a desired detector configuration, for example, by both ultraviolet (UV) and evaporative light scattering. These detectors provide real-time feedback on the elution of polymer fractions, allowing researchers to monitor and adjust the separation process as needed. An evaporative light scattering detector (ELSD) is especially useful for monitoring the elution profile of polymeric materials with minimal or no UV absorption. Additionally, chromatograms, sample collection, and detector output can be automatically recorded to enable data interpretation, visualization, and comparison among experiments. Fractions are automatically collected in preweighed test tubes (e.g., 16 × 150 mm test tubes, 22 mL increments) and evaporated to dryness and weighed to determine the final masses. The use of automation and appropriate column conditions allows for high mass recoveries. How fine a separation is achieved can be easily controlled by changing the size of each test tube. Fractions with similar compositions can also be carefully combined as desired for further structural characterization.

## 3 Rapid Access to Discrete Oligomer Libraries

**Figure 3:**
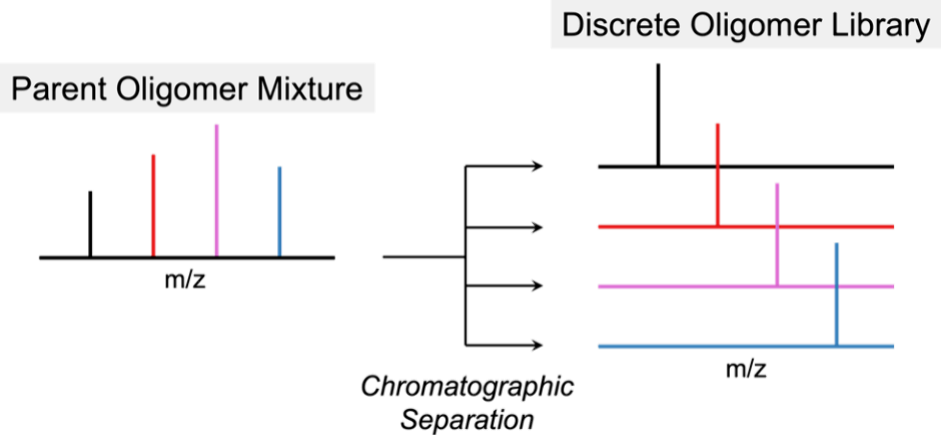
Multigram chromatographic separation of oligomeric mixtures prepared by controlled polymerization affords discrete oligomers.

Oligomers are low-molecular-weight polymers with the International Union of Pure and Applied Chemistry (IUPAC) defining an oligomer as “a molecule of intermediate relative molecular mass which has properties which do vary significantly with the removal of one or a few of the units” (emphasis added).^44^ Consequently, the impact of molecular weight dispersity is exacerbated in oligomers due to the small number of repeating units present, highlighting the potential opportunities for general scalable strategies to monodisperse synthetic oligomers for targeted applications. For example, a low-dispersity oligomer (degree of polymerization (DP) ≈ 8, *Đ* = 1.2) prepared using controlled polymerization was found to be primarily a mixture of species ranging from DP = 4 to DP = 12 with chains of DP = 8 comprising <15 mol % of the mixture through chromatographic fractionation. As implied by IUPAC, the material properties of oligomeric materials are sensitive to small changes in chain length, sequence, and stereochemistry with the preparation of precise oligomeric materials providing an opportunity to understand fundamental structure–property relationships. To circumvent the challenges associated with iterative exponential growth strategies, our group has leveraged automated chromatographic separation with controlled polymerization techniques to prepare a wide variety of discrete oligomers, thereby allowing nonexperts to rapidly generate well-defined oligomer libraries (Figure 3).

**Figure 4:**
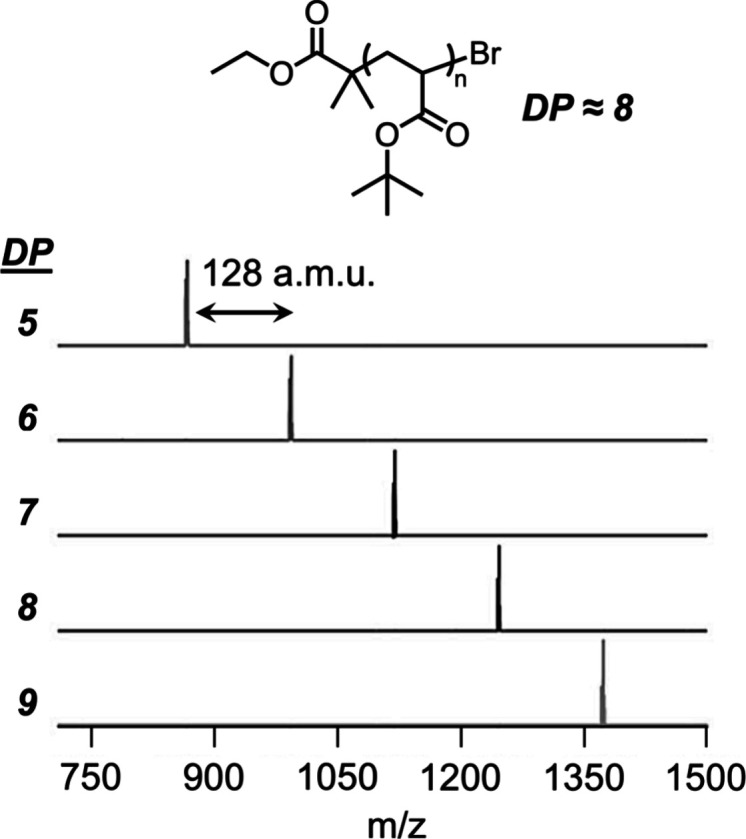
MALDI spectra of discrete oligo(*tert*-butyl acrylate) with DP = 5–9 isolated after a multigram chromatographic separation of an as-synthesized DP ≈ 8 mixture. Reproduced with permission from ref (1). Copyright 2016 American Chemical Society.

To demonstrate the versatility in separating discrete oligomers from disperse parent materials, representative low-molecular-weight poly(*tert*-butyl acrylate) homopolymers (DP_NMR_ ≈ 8, *Đ*_SEC_ = 1.2) were synthesized by ATRP on a 50–100 g scale.^1^ The parent material was loaded on a multigram scale (15 g) onto a normal-phase column cartridge and separated using an optimized hexanes/ethyl acetate gradient from 100% hexanes to 25 vol % ethyl acetate in hexanes. While the parent material had an average DP of 8 by ^1^H nuclear magnetic resonance (NMR), discrete oligomers from trimers to decamers were isolated (1–3 g each) after the separation, with shorter oligomers eluting first, and an excellent mass recovery of discrete materials of ∼50% was observed. As evidenced by matrix-assisted laser desorption/ionization–mass spectrometry (MALDI-MS), single molecular ions were observed for each discrete oligomer separated by 128 amu, corresponding to the *tert*-butyl acrylate repeat unit (Figure 4). Importantly, this technique was shown to reproducibly fractionate poly(*tert*-butyl acrylate) oligomer mixtures with a variety of starting average degrees of polymerization as well as distinct chain ends (e.g., *tert*-butyl initiator, alkyne initiator, or trithiocarbonate) without the need to modify the optimized solvent gradient. The physical properties of these discrete oligomers were found to depend on the degree of polymerization, with significant differences observed relative to the parent material. While the initial as-synthesized polymer was a viscous oil, discrete oligomers above DP = 4 were obtained as solid waxes, with the glass transition temperature increasing with the degree of polymerization from −30 to 0 °C. These results underscore the pronounced impact of oligomer length and dispersity on material properties and the importance of discrete materials in understanding fundamental structure–property relationships.

The ability to fractionate poly(*tert*-butyl acrylate) oligomer mixtures with a variety of end groups was leveraged to elucidate the molecular factors that impact the performance of polymeric ^19^F magnetic resonance imaging (MRI) agents.^45^ In this study, a fluorinated chain-transfer agent was used in the RAFT polymerization of *tert*-butyl acrylate, leading to oligomers with a single CF_3_ chain end. Deprotection of the *tert*-butyl esters with trifluoroacetic acid yielded discrete oligo(acrylic acid), each bearing a single CF_3_ end group for ^19^F MRI agents. Importantly, both the oligomer length and dispersity were determined to have a strong effect on magnetic resonance performance, where the signal-to-noise ratio systematically increased with the degree of polymerization. This work provides a powerful strategy for understanding the design principles that govern the efficacy of ^19^F MRI oligomeric imaging agents.

**Figure 5:**
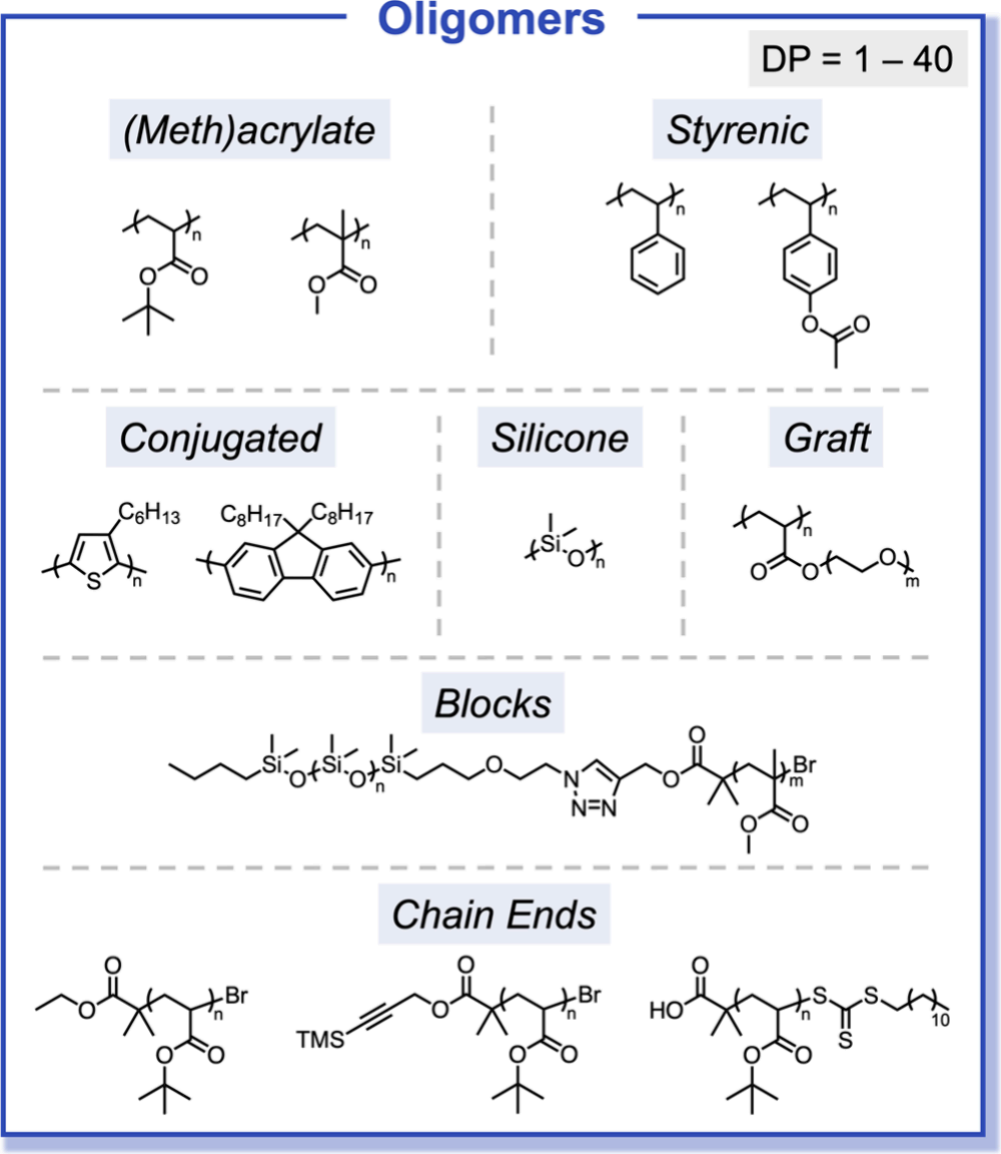
Chromatographic separation enables scalable access to discrete oligomers across a broad range of monomer families, degrees of polymerization, chain-end functionalities, and architectures.

**Figure 6:**
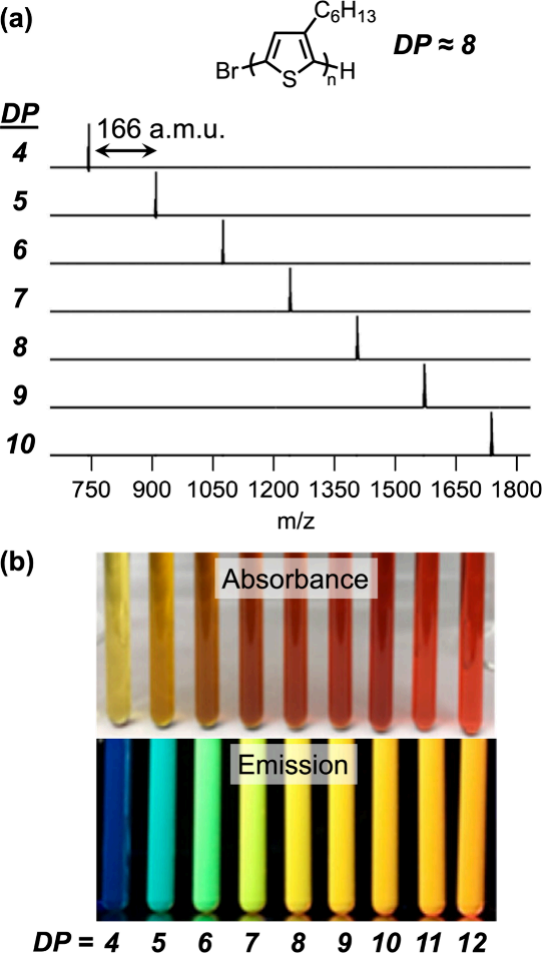
(a) MALDI spectra of discrete oligo(3-hexylthiophene), DP = 4–10, isolated after multigram chromatographic separation of an as-synthesized DP ≈ 8 mixture. (b) Discrete oligo(3-hexylthiophene) exhibits chain-length-dependent optoelectronic properties below the effective conjugation length. Reproduced with permission from ref (2). Copyright 2017 American Chemical Society.

An advantage of standard chromatography strategies is preparing discrete oligomers across a wide range of monomer families, including acrylates, methacrylates, styrenics, and siloxanes.^1^ The ability to carefully control separation conditions is enabling in the context of broadly separating materials with diverse chemistries, chain lengths, and architectures (Figure 5). Further expanding on our initial oligomer substrate scope, we extended the application of automated chromatography to the preparation of discrete conjugated oligomers.^2^ Conjugated polymers are an important class of materials due to their unique optoelectronic properties with applications ranging from advanced organic electronics to nanoscale assemblies.^46^ The influence of dispersity becomes particularly pronounced in the context of conjugated oligomeric materials given the low degrees of polymerization that closely align with the effective conjugation length.^47^ Below the effective conjugation length, conjugated materials exhibit optical properties that are strongly dependent on the degree of polymerization. To demonstrate the ability to rapidly prepare discrete conjugated oligomers from a single parent sample, a parent oligo(3-hexylthiophene) polymer was separated, and MALDI-MS analysis of the fractionated samples revealed elution of precise oligothiophenes ranging from discrete trimer to dodecamer chains (Figure 6a). Importantly, as the degree of polymerization of 3-hexylthiophene oligomers increases, a systematic red shift in the absorbance and emission spectra maxima was observed, eventually converging for the dodecamer DP = 10 (Figure 6b). The power of this approach is further highlighted through its broad utility with other conjugated oligomers, including oligo(fluorene)s and oligomeric trithiophene derivatives.

An intriguing opportunity with discrete oligomers is their use as building blocks to construct more complex and defined materials. One example is block co-oligomers with tailored molar mass dispersities. Discrete (*Đ* = 1.0) and near-discrete (*Đ* ≈ 1.05) block co-oligomers were prepared by coupling separately fractionated homo-oligomers to understand the impact of dispersity on self-assembly.^48^ Oligomers of dimethylsiloxane and methyl methacrylate were chosen as a model system due to their high Flory–Huggins interaction parameter (*χ*_*ij*_), which enables microphase separation at low degrees of polymerization. Discrete oligo(dimethylsiloxane) samples were prepared through reverse-phase column chromatography on C_18_ silica gel with a methanol/hexanes eluent, while oligo(methyl methacrylate) was separated via normal-phase chromatography using an optimized acetonitrile/toluene gradient. By accurately controlling chain ends, oligomers with varied levels of dispersity could be coupled together through copper-mediated click chemistry to form a wide range of block co-oligomers. Small-angle X-ray scattering (SAXS) experiments revealed that discrete block co-oligomers have smaller domain spacings and sharper scattering reflections compared to disperse analogues. Significantly, the order–disorder transition temperature (*T*_ODT_) was found to decrease with increasing dispersity. These results highlight the powerful effect of subtle changes in dispersity on block copolymer self-assembly.

**Figure 7:**
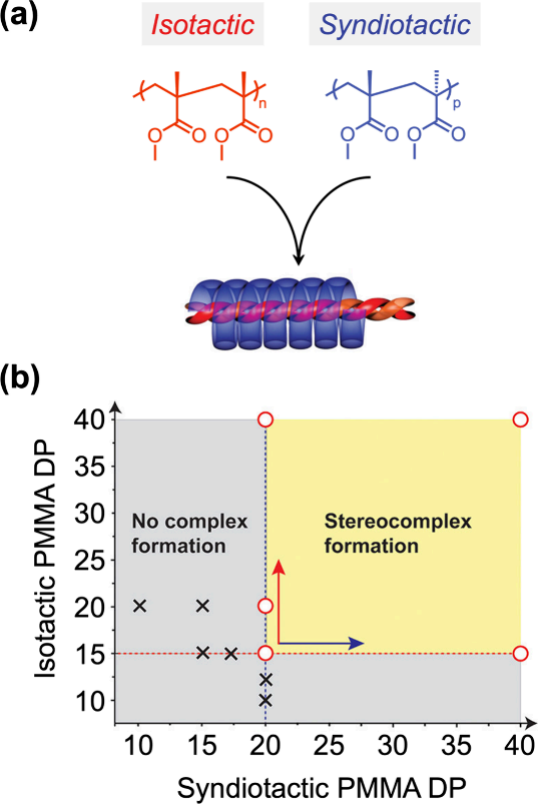
(a) Schematic illustration of the triple-helix stereocomplex of isotactic and syndiotactic PMMA. (b) Chromatographic separation elucidated the critical chain lengths needed to form a triple-helix PMMA stereocomplex. Reproduced with permission from ref (50). Copyright 2018 American Chemical Society.

The ability to prepare discrete oligomers creates unique opportunities related to other self-assembly phenomena as well. One example is the supramolecular assembly of isotactic (*it*) and syndiotactic (*st*) poly(methyl methacrylate) (PMMA), which are known to form a triple-helix structure with a double-stranded inner helix of *it*-PMMA wrapped by a single-stranded outer helix of *st*-PMMA (Figure 7a).^49^ To fully understand the critical chain length required for stereocomplex formation, a combination of stereospecific polymerization and automated chromatography was used to generate discrete *it*-PMMA and *st*-PMMA oligomers.^50^ These novel discrete oligomers were combined to identify the minimum degree of polymerization required for stereocomplex formation, determined to be precisely a 15 mer *it*-PMMA and a 20 mer *st*-PMMA (Figure 7b). The unprecedented availability of these discrete building blocks allowed the self-sorting of these complexes to generate thermodynamically stable structures, reminiscent of DNA, to be studied. This result underscores the utility of precise materials in connecting molecular design with crystallization behavior and offering new insights into complex molecular assembly.

Drawing an analogy with biomacromolecules, the isolation of discrete and sequence-specific synthetic macromolecules could have significant implications in biological and pharmaceutical research. One appealing target, poly(ethylene glycol) (PEG), features prominently in therapeutics and drug delivery^51−53^ as exemplified by 1,2-dimyristoyl-rac-glycero-3-methoxypolyethylene glycol-2000 (DMG-PEG2000). This critical component in the Moderna COVID-19 vaccine involves a single lipid group coupled to a disperse PEG chain having an average molecular weight of 2000 g/mol.^54^ Because the PEG chain is disperse, small batch-to-batch variations may exhibit different behavior in the body. Understanding the role of such dispersity would yield crucial insights into the safety and biodistribution profile of PEG in biological applications. Motivated by this goal, our group developed a scalable and versatile strategy to prepare discrete PEG libraries.^55^ Low-molecular-weight homopolymers of both tri- and tetra(ethylene glycol) acrylate were synthesized from a lipid-based ATRP initiator and separated to prepare gram-scale discrete lipid-PEG libraries using a hexanes/ethyl acetate/tetrahydrofuran mixed solvent gradient. The utility of ATRP in this strategy was demonstrated by the presence of a synthetically accessible, single bromo chain end retained on the discrete copolymers that can be replaced by hydrogen through Pd-catalyzed hydrogenation or converted to the corresponding azido group with NaN_3_.^56,57^ Dynamic light scattering experiments reveals an increase in the lower critical solution temperature (LCST) with increasing overall ethylene glycol units. Notably, discrete oligomers with hydrogenated chain ends exhibit different LCST behavior than their bromo counterparts, highlighting the pronounced influence of precise chain ends, oligomer length, and overall number of ethylene glycol units on material properties. Significantly, the discrete branched copolymers were shown to promote efficient nanoparticle assembly while reducing anti-PEG antibody recognition when compared to commercial polydisperse DMG-PEG2000. This result underscores the enhanced potential of precisely engineered materials for improving performance in biological applications.

The accessibility of automated chromatography has facilitated its adoption by an increasing number of research groups around the world. Junkers and colleagues have a significant body of work using automated chromatographic separation for the preparation of higher-order sequence-defined discrete oligomers, including oligoacrylates^58−61^ and nucleobase-containing oligomers.^62^ Gibson and colleagues demonstrated the critical chain length required for inhibiting biomimetic ice recrystallization through the preparation of discrete vinyl alcohol oligomers.^63^ In a similar vein, Zhu and colleagues determined the chain length effects on the photophysical and crystalline properties of discrete conjugated oligo(fluorenediacetylene).^64^ Bonilla-Cruz and co-workers prepared a series of monodisperse oligo(δ-valerolactone) and oligo(ε-caprolactone) through chromatographic fractionation, highlighting that crystallinity is directly proportional to chain length.^65^ Finally, Miura and colleagues demonstrated that the affinity to and sequence specificity of oligomeric ligands to target peptides are strongly dependent on the number of functional groups.^66^ Collectively, the large body of work summarized in this section highlights the versatility of automated chromatography in the scalable synthesis and study of discrete oligomers.

## 4 Rapid Generation of Block Copolymer Libraries

Block copolymers are an important class of materials that self-assemble into a rich array of nanoscale morphologies. The distinct ability of block copolymers to spontaneously self-assemble into well-defined nanostructures underpins their versatility, enabling applications in advanced separation membranes, thermoplastic elastomers, photonic crystals, microelectronics, and drug delivery.^67−71^ Key to these and other applications is the ability to tune self-assembly through synthetic handles including block chemistry, block sequence, composition, molecular weight, and dispersity using controlled polymerization techniques. This long list of structural variables illustrates the difficulty in navigating and controlling a multidimensional design space, underscoring the potential advantages of developing an accelerated approach to the generation of high-purity, well-defined block copolymer libraries. Traditional methods of constructing even incomplete block copolymer phase diagrams involve iterative synthesis followed by multiple purification and isolation steps, greatly increasing the time and cost of materials discovery. The repetitive synthesis of multiple block copolymers is also complicated by slight variations in reaction conditions and/or purification that lead to undesired (but unavoidable) differences among samples and the presence of variable amounts of homopolymer impurities. As a result, the current gold standard of individually synthesizing modest-sized libraries of block copolymers represents a rate-limiting step in the study of these materials both commercially and scientifically (Figure 9a).

**Figure 8:**
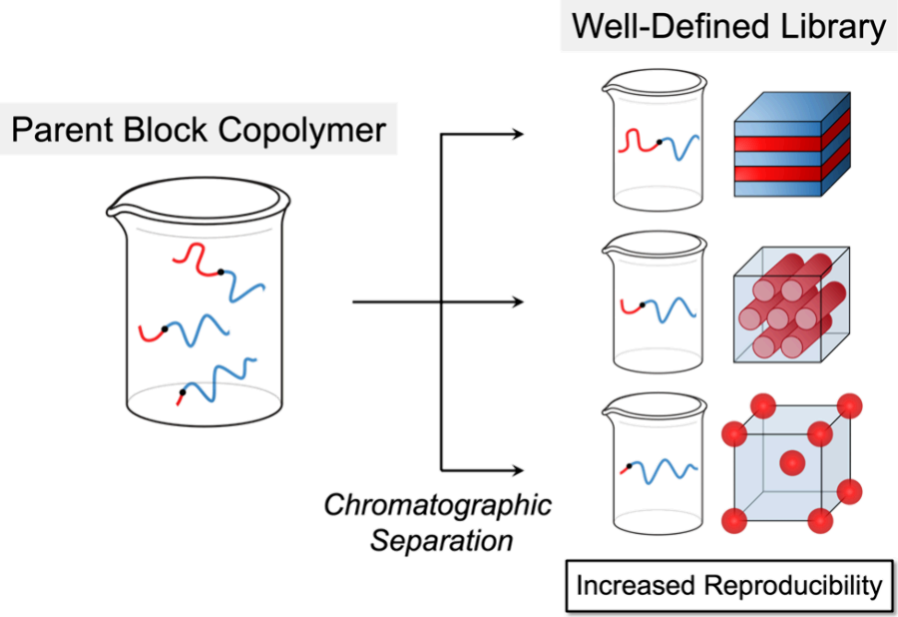
Automated chromatographic separation of as-synthesized block copolymers generates well-defined libraries of fractionated samples spanning a wide range of compositions and morphologies. Fractionation also removes impurities such as homopolymers that are generated during synthesis, which improves reproducibility.

**Figure 9:**
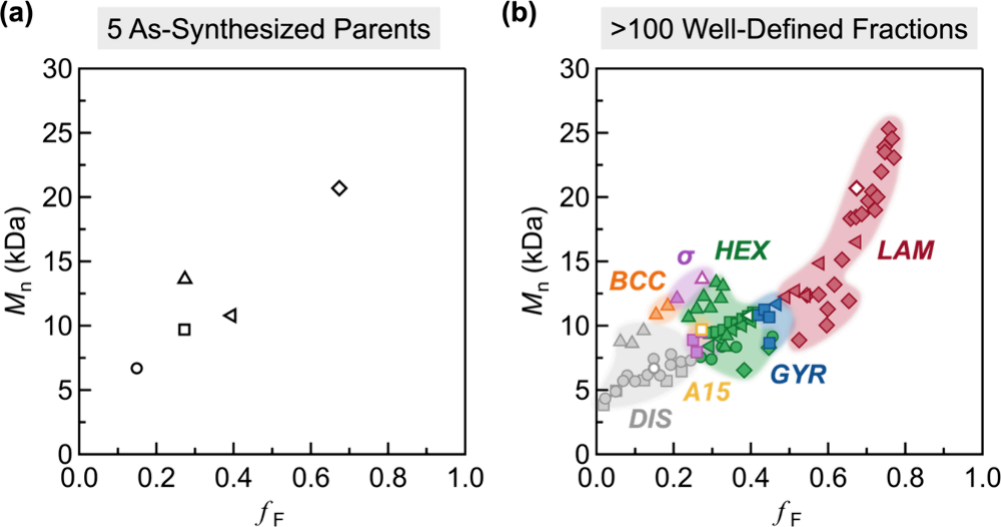
(a) Limited phase diagram from five as-synthesized, parent block copolymers prepared through iterative synthesis. (b) Comprehensive phase diagram from a library of >100 well-defined, fractionated block copolymers generated via chromatographic separation of five parent materials. Reproduced with permission from ref (74). Copyright 2024 American Physical Society.

Building on the successful scalable synthesis of discrete oligomers, automated chromatography is also a powerful technique in the context of separating as-synthesized block copolymers into libraries of well-defined and purified materials (Figure 8). This versatile, scalable, and accessible method greatly facilitates the isolation of block copolymers with tailored molecular weights, molar mass dispersities, compositions, segregation strengths, and architectures to build comprehensive phase diagrams from a minimal number of syntheses, thereby accelerating the study of structure–property relationships in advanced soft materials (Figure 9b).

**Figure 10:**
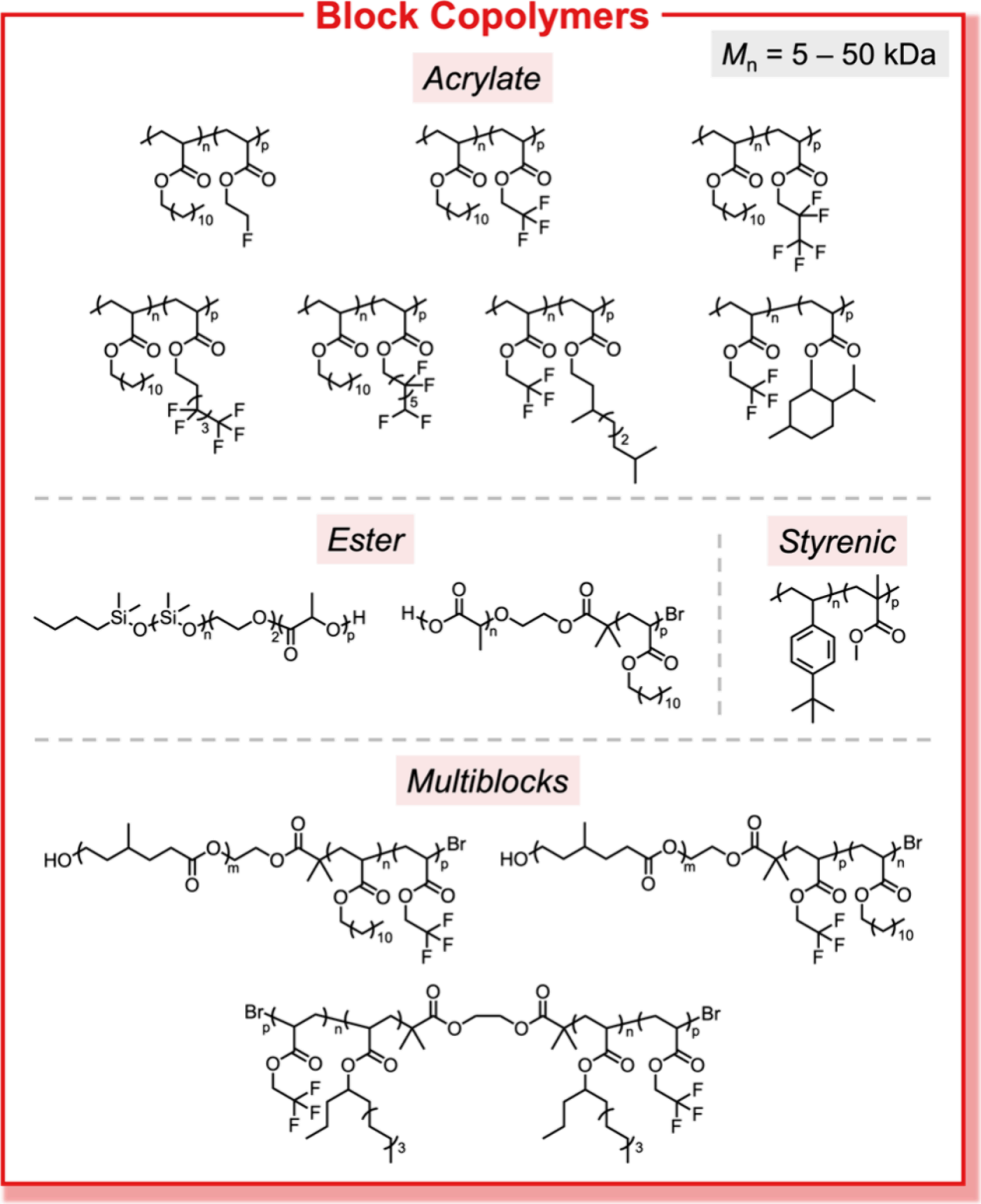
Chromatographic separation enables efficient and scalable access to well-defined block copolymer libraries across a broad range of monomer families, molecular weights, compositions, architectures, and block sequences.

To highlight the efficiency of automated chromatography in rapidly generating block copolymer libraries, our initial studies focused on separating prototypical AB diblock copolymers. Since previous work demonstrated the rich phase behavior of poly(dodecyl acrylate)-*b*-poly(lactide) (PDDA-*b*-PLA) through the individual synthesis of 13 different diblocks,^72^ automated chromatography was applied to the same system to determine the phase behavior from separating a single parent PDDA-*b*-PLA sample compared to the traditional multisynthesis strategy. A library of 20 well-defined diblock copolymers, spanning a broad range of compositions, was readily prepared in 1 h from a single parent block copolymer to prepare an enhanced phase diagram.^73^ An added benefit of this separation process is the removal of homopolymer impurities and a decrease in the dispersity of each fraction compared to the parent block copolymer, highlighting the improved purity of the fractionated samples. The sequence of phases matched the results obtained from iterative synthesis with important differences in order–order phase boundaries that may be related to differences in dispersity and purity for the fractionated samples. Automated chromatography was similarly found to be useful for a broad range of other monomer pairs, including high-*χ* poly(dimethylsiloxane)-*b*-poly(lactide) and poly(4-*tert*-butylstyrene)-*b*-poly(methyl methacrylate) as well as a family of poly(trifluoroethyl acrylate)-based conformationally asymmetric materials (Figure 10).

**Figure 11:**
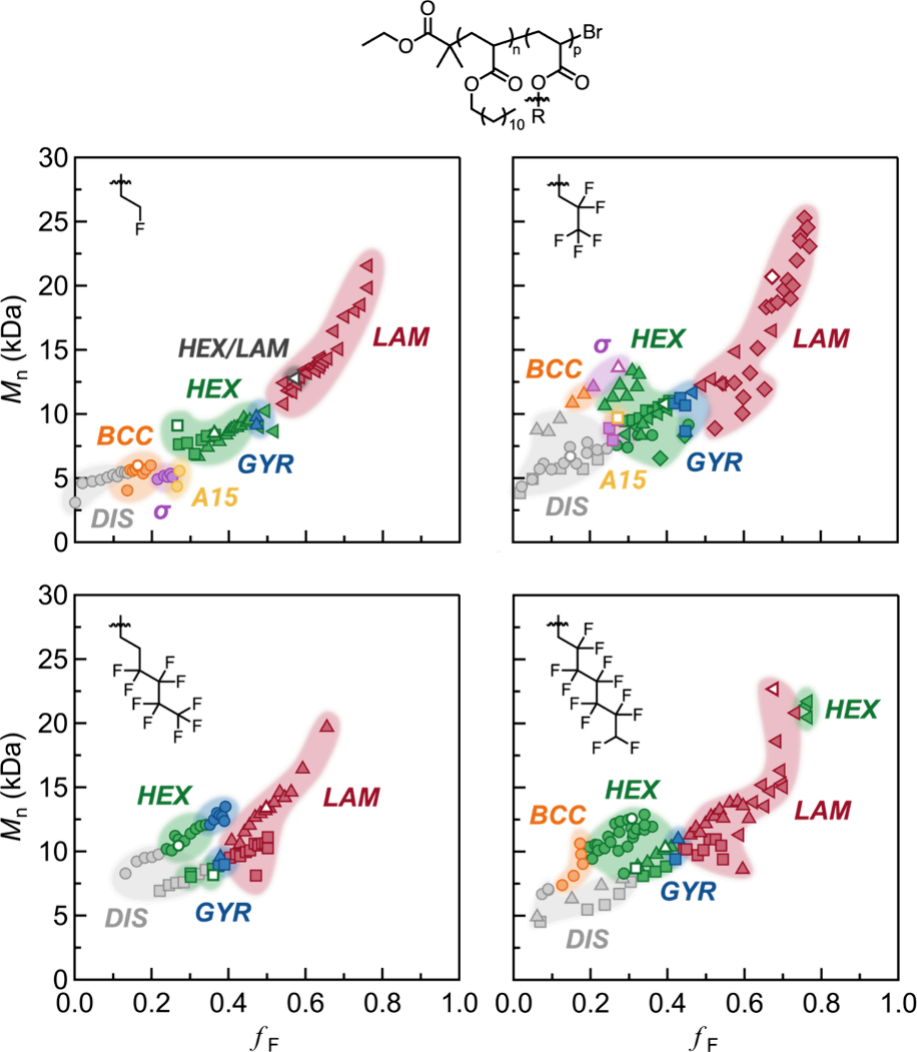
A library of >320 well-ordered diblock copolymers derived from the synthesis and separation of only 16 samples enabled the preparation of four comprehensive phase diagrams with a high degree of compositional resolution. As-synthesized parent materials are depicted with an open symbol. Fractionated diblock copolymers are depicted with the same shape as their respective parent material but filled. Color indicates the morphology as determined by small-angle X-ray scattering. Reproduced with permission from ref (74). Copyright 2024 American Physical Society.

Because of the significant acceleration in discovery provided by automated chromatography, it is particularly powerful in efficiently mapping out phase diagrams of distinct block copolymer chemistries. Recent work has leveraged this concept in understanding the phase behavior of diblock copolymers having varying degrees of fluorination.^74^ Samples with 1 to 12 fluorine atoms per monomer unit as one of the blocks were fractionated on multigram scales using similar separation protocols with excellent mass recoveries (>80%). Remarkably, over 300 purified and well-defined diblock copolymers were prepared from the synthesis and separation of only 16 parent samples. The power of this separation strategy in preparing detailed phase diagrams is enhanced by an ability to rapidly and precisely identify order–order boundaries and morphologies with extremely narrow windows of stability (∼1 vol %) such as gyroid, which was consistently found in all four phase diagrams (Figure 11). Moreover, both composition and domain spacings can be finely tuned with angstrom-level resolution. This level of precision is challenging to achieve using traditional iterative synthesis or conventional polymer purification strategies but has significant potential for lithographic and optical applications.

**Figure 12:**
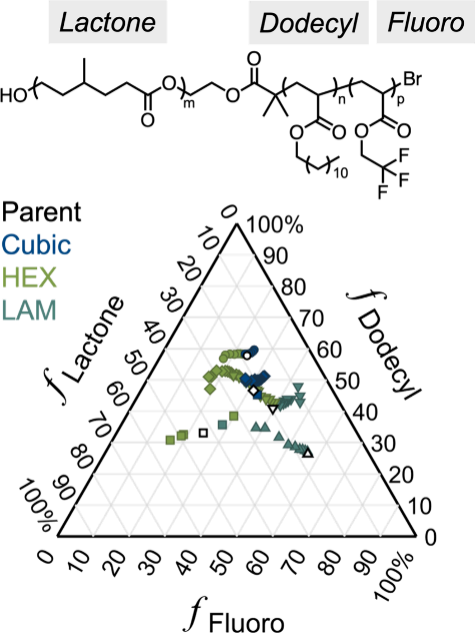
A library of ∼100 well-defined, fractionated ABC triblock terpolymers generated via automated chromatographic separation. Fractionated triblock terpolymers are depicted with the same shape as their respective parent material but with filled symbols. Color indicates the morphology as determined by small-angle X-ray scattering. Reproduced with permission from ref (4). Copyright 2022 American Chemical Society.

While the simplest block copolymer sequence, an AB diblock copolymer, has been extensively studied by a synergistic combination of experiments and simulations, similar studies are still needed to provide a comprehensive picture of multiblock copolymer phase behavior.^69^ Multiblock copolymers with increasingly complex block sequences—for example, ABC triblock terpolymers—offer unique opportunities to create nanostructured materials, but this potential has been hampered by an even larger design space that complicates the exploration of structure–property relationships.^75^ To overcome these challenges, we applied automated chromatography to generate over 100 purified narrow-dispersity well-ordered triblock terpolymers from just eight as-synthesized ABC and isomeric ACB parent samples.^4^ To demonstrate the utility of automated chromatography, 1.5 g of a parent triblock terpolymer was dissolved in dichloromethane and loaded directly onto a commercially available (100 g) silica gel column to give 30 purified triblock samples (30–60 mg each). Significantly, an overall mass recovery of 90% was achieved for this multigram-scale separation. Homopolymer and diblock copolymer impurities were readily identified and removed due to their elution at the beginning of the fractionation process. While the as-synthesized parent triblock was ordered, a definitive morphology was difficult to determine. In contrast, the fractionated samples had well-defined structures with exceptional long-range order, as evidenced by upward of 20 reflections observed in many SAXS patterns. Notably, through the synthesis of >10 parent triblock terpolymers, a ternary phase diagram consisting of >100 ABC and isomeric ACB fractions with well-defined morphologies was generated, illustrating the significant acceleration in discovery that is afforded by automated chromatography (Figure 12).

Recent advances in controlled polymerization techniques have revolutionized the synthesis of intricate polymer sequences and architectures. However, conventional synthetic approaches for the preparation of complex materials suffer from inevitable difficulties in fully preventing the formation of homopolymer or diblock copolymer impurities, which are traditionally challenging to even identify, let alone remove. As mentioned above, automated chromatography *does* allow for the detection and, importantly, removal of small amounts of homopolymer impurities (<2%) generated during synthesis. This power of impurity identification and removal is particularly acute in the study of block copolymer phase behavior, as homopolymer contamination has been shown to impact self-assembly in a nontrivial way.^76,77^ Our group demonstrated a surprisingly pure hexagonally close-packed (HCP) sphere phase formed by linear block copolymers over a wide range of compositions.^78^ The HCP morphology was identified in ABA triblocks and AB diblocks. To support the purity of the as-synthesized HCP-forming materials, a triblock copolymer with HCP morphology was fractionated into 10 purified samples, where no evidence of homopolymer was identified in any fraction. Significantly, automated chromatography helped support the inference that HCP can be accessed in linear block copolymer melts without the use of blending or other complex processing techniques.

Automated chromatography has similarly found widespread use in the rapid exploration of structure–property relationships for other complex materials. Lawrence and colleagues prepared discrete bottlebrushes and discovered marked differences in monolayer phase transitions, glass transition temperatures, and packing efficiency compared to disperse materials.^79,80^ Smith and co-workers demonstrated the role of both side-chain length and dispersity on gas transport properties and plasticization resistance in bottlebrush polymers.^82^ In a similar vein, Benetti and colleagues investigated the effect of side-chain dispersity in surface-grafted polymer brushes, elucidating the importance of dispersity for antifouling applications.^81^

## 5 Conclusions and Perspectives

This Account demonstrates the power and potential of automated chromatographic fractionation, often coupled with controlled polymerization, for accessing discrete and well-defined polymer libraries. The simplicity, versatility, and scalability of the separation strategy overcomes many of the current challenges and drawbacks associated with serial synthesis and other post-polymerization purification strategies. We hope readers are inspired by the broad applicability and availability of this technique to non-experts, for example, in isolating discrete oligomers or expansive sets of block copolymers, and see opportunities to adopt it in their own research. Whether the goal is answering fundamental questions or carefully tailoring structure–property relationships for a specific application, automated chromatography is poised to provide a significant return on effort by accelerating discovery and fostering new possibilities in science and engineering.

Looking forward, the future is bright. We fully anticipate that the concepts outlined above will be transferable to other classes of soft materials of contemporary importance, such as more complex multiblocks, bottlebrushes, and stars. Perhaps more excitingly, it is interesting to ponder whether the speed and simplicity of chromatographic separation might prove transformative when integrated with machine learning, artificial intelligence (AI), and/or complementary types of automated tools. With synthesis as a common bottleneck in discovery workflows, the potential for creating very large high-quality data sets and sample libraries for training AI tools certainly provides a pathway to bridge the divide between traditional big data and experimental materials science. We, and many others in the community, anticipate that these new research directions will lead to exciting developments in the years to come.
